# Uptake and anti-inflammatory effects of liposomal astaxanthin on endothelial cells tracked by Raman and fluorescence imaging

**DOI:** 10.1007/s00604-023-05888-8

**Published:** 2023-07-27

**Authors:** Basseem Radwan, Amrutha Prabhakaran, Stefano Rocchetti, Ewelina Matuszyk, Tia E. Keyes, Malgorzata Baranska

**Affiliations:** 1 Jagiellonian Centre for Experimental Therapeutics (JCET), Jagiellonian University, 14 Bobrzynskiego Str., 30-348 Krakow, Poland; 2 Faculty of Chemistry, Jagiellonian University, 2 Gronostajowa Str., 30-387 Krakow, Poland; 3 School of Chemical Sciences and National Centre for Sensor Research, Dublin City University, Dublin 9, Ireland

**Keywords:** Endothelium, Carotenoids, Liposomes, Cellular uptake, Raman microscopy

## Abstract

**Abstract:**

Astaxanthin (AXT) is a lipophilic antioxidant and anti-inflammatory natural pigment whose cellular uptake and bioavailability could be improved via liposomal encapsulation. Endothelial cells (EC) line the lumen of all blood vessels and are tasked with multiple roles toward maintaining cardiovascular homeostasis. Endothelial dysfunction is linked to the development of many diseases and is closely interconnected with oxidative stress and vascular inflammation. The uptake of free and liposomal AXT into EC was investigated using Raman and fluorescence microscopies. AXT was either encapsulated in neutral or cationic liposomes. Enhanced uptake and anti-inflammatory effects of liposomal AXT were observed. The anti-inflammatory effects of liposomal AXT were especially prominent in reducing EC lipid unsaturation, lowering numbers of lipid droplets (LDs), and decreasing intercellular adhesion molecule 1 (ICAM-1) overexpression, which is considered a well-known marker for endothelial inflammation. These findings highlight the benefits of AXT liposomal encapsulation on EC and the applicability of Raman imaging to investigate such effects.

**Graphical Abstract:**

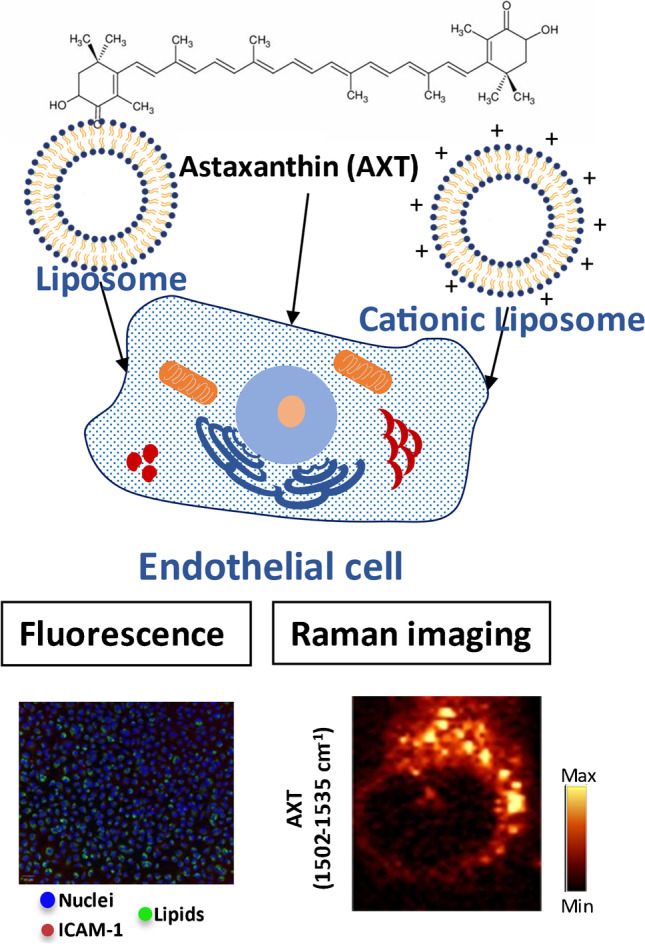

**Supplementary Information:**

The online version contains supplementary material available at 10.1007/s00604-023-05888-8.

## Introduction

Liposomal encapsulation has found a wide range of applications including its use to solubilize, increase bioavailability, and improve the stability of several bioactive molecules and natural products [1, 2]. One example of such molecules is astaxanthin (AXT). AXT is a naturally occurring red pigment that has been featured in multiple studies for its antioxidant and anti-inflammatory effects; it has the potential to suppress cancer and can act as an antidiabetic agent [3–7]. Due to its chemical structure, as a carotenoid that consists of a long polyene chain and two terminal rings, AXT is highly lipophilic, which limits its bioavailability and consequently its use as an antioxidant dietary supplement. Liposomal encapsulation is one of the methods shown to improve AXT bioavailability, antioxidant effect, stability, and solubilization. It was reported that both neutral and positively charged liposomes enhance the efficacy of AXT cytoprotective effects in vitro [8–10].

Endothelial cells (EC) form a monolayer, lining the lumen of all blood vessels in the body, and participate in multiple activities involved in maintaining cardiovascular homeostasis [11]. EC play an important role in the regulation of blood flow, vascular permeability, thrombosis, angiogenesis, and inflammatory response [12–15]. Endothelial dysfunction (ED) occurs as a result of the impairment of EC regulatory functions and is linked to the development of many diseases including atherosclerosis, hypertension, liver diseases, diabetes, and even COVID-19 [11, 16–20]. Furthermore, the development of many ED-related pathologies is closely linked to oxidative stress and vascular inflammation [14].

One of the widely used in vitro models of endothelial inflammation is the stimulation of EC by tumor necrosis factor alpha (TNF-α). Upon incubation with TNF-α, EC are activated through the nuclear factor kappa-light-chain-enhancer of activated B cells (NF-κB) pathway [21, 22]. This leads to increased expression of leukocyte adhesion molecules such as intercellular adhesion molecule 1 (ICAM-1), vascular cell adhesion molecule 1 (VCAM-1), and cyclooxygenase-2 (COX-2), and alterations in EC lipid content, specifically lipid droplets (LDs) [23, 24]. LDs are lipid-rich cellular organelles that not only act as energy reservoirs but also play an important role in regulating the uptake, metabolism, distribution, and utilization of lipids in cells. Moreover, abnormalities in the content and structure of LDs have been recognized as markers for pathogenesis in cells [25, 26]. For instance, inflamed EC have been shown to contain significantly more LDs of unsaturated lipids compared to control EC [22].

One of the powerful tools used to study cells and their content is Raman microscopy. This method allows clear detection of the subcellular lipids and provides valuable information on their chemical composition in a label-free manner by following the Raman bands associated with lipids in the following spectral regions: 3000–2800 cm^−1^ (C–H stretching), 1500–1400 cm^−1^ (CH_2_ group scissoring), 1300–1250 cm^−1^ (CH_3_ group twisting), and 1200–1050 cm^−1^ (C–C stretching) [25, 27–29]. Nevertheless, Raman imaging of biomolecules, including lipids, can be improved using molecular Raman probes (Rp) [25, 30, 31]. We previously introduced astaxanthin as a resonance Raman probe for lipids in EC, showing its potential in signaling subcellular lipidic structures, such as LDs, using relatively lower laser power than required for label-free Raman imaging [26, 32].

AXT absorbs the light in the visible range that coincides with the excitation wavelength of lasers commonly used for cell analysis. AXT fluorescence analysis demonstrates its emission maximum in solution to be around 570 nm [33]; however, the fluorescence emission of AXT depends on its environment, as 600 ± 40 nm was used to analyze AXT cellular content [34, 35]. The Raman spectrum of AXT, when excited by a 532 nm laser, consists of resonantly enhanced bands at ca. 1520, 1159, and 1009 cm^−1^ [26, 32]. This noticeable signal enhancement allows for the detection of AXT in cells and makes it possible to follow AXT cellular uptake using Raman imaging.

In this study, we aim to follow EC uptake of free and encapsulated AXT using Raman microscopy. Furthermore, we aim to investigate the effects of AXT and AXT-loaded liposomes on inflammatory EC and the resulting alterations in lipid composition. This will shed the light on the potential of bimodal (Raman and fluorescence) imaging to investigate cellular uptake and anti-inflammatory effects of relatively small molecules in free and encapsulated forms.

## Materials and methods

### Preparation of astaxanthin-loaded neutral and cationic liposomes

Neutral and cationic liposomes were prepared by hydration extrusion method followed by the reconstitution of (3S,3′S)-astaxanthin (AXT, Sigma Aldrich). DPPC (1,2-dipalmitoyl-sn-glycero-3-phosphocholine) lipid was used for the preparation of neutral liposomes and a mixture of DOPE/DOTAP (1,2-dioleoyl-sn-glycero-3-phosphoethanolamine and 1,2-dioleoyl-3-trimethylammonium-propane) was used for cationic liposome preparation. AXT in DMSO was added directly to the liposomes to a final concentration of 10 μM in the liposome solution (molar ratios were as follows: DPPC:AXT = 1:0.007 and DOPE:DOTAP:AXT = 1:0.98:0.036). Chloroform solution of the lipid was dried under nitrogen flow to get a lipid film in a 1.5-mL glass vial and it was kept under high vacuum for 30 min to ensure the complete removal of the solvent. The lipid film was hydrated with 1 mL phosphate buffer saline of pH 7.4 and vortexed for 60 s to get the suspension of lipid in the buffer. This suspension was extruded at least 11 times through a polycarbonate membrane of 100 nm pore size. AXT (10 μM) was reconstituted into the liposomes by 30 min incubation. When AXT was added to the lipid before extrusion, it was observed that the AXT is not getting encapsulated into the liposomes fully as it was clearly visible in the polycarbonate membrane. Different concentrations of AXT ranging from 0.5 to 10 μM were tried with various ratios of lipids before arriving to the optimal ratios (mentioned above) for AXT liposomal encapsulation and cell measurements. Experiments based on 1 μM AXT liposomal concentration (final concentration of 0.33 μM in cell culture media) did not show promising results during the cell-based experiments. Best results were obtained with 10 μM AXT was added to the liposomes after the extrusion and the unbound AXT was removed by dialysis. Any unbound AXT was removed by 5 h of dialysis using pur-A-lyzer kit. DPPC was extruded at 45 °C, above the phase transition temperature.

The hydrodynamic diameters of the samples were measured by dynamic light scattering (DLS) on Malvern Zetasizer Ultra. UV-vis spectra were measured using Jasco V670 UV/Vis spectrophotometer, and fluorescence was measured using a Varian Cary Eclipse Fluorescence spectrophotometer in a quartz cuvette of 1 cm pathlength.

### Cell culture and MTS assay

For subsequent Raman measurements, primary human aortic endothelial cells (HAoEC) were seeded on calcium fluoride (CaF_2_) slides, and for fluorescence imaging, cells were seeded on glass-bottom 96-well plates to reach their optimal confluency after 24 h. HAoEC were cultured in a complete EGM-2MV medium (Lonza, Basel, Switzerland) that had the following supplementations: 10 mM L-glutamine (Gibco Life Technologies), 1 μg/mL hydrocortisone (Sigma Aldrich), 10 mg/mL epidermal growth factor (EGF, Sigma Aldrich), 10% fetal bovine serum (FBS, Gibco Life Technologies), and 1% of antibiotics (streptomycin, penicillin, and fungison, Gibco Life Technologies). All cells were incubated in a 37 °C, 5% CO2/95% air humidified cell culture incubator. Cells were either pre-treated with 10 ng/mL human tumor necrosis factor alpha (TNF-α, Sigma Aldrich) or kept in a fresh medium for 24 h. Afterward, cells (except for the control group) were treated with cell culture media containing 1 μM AXT (Sigma Aldrich), dilution of liposome solutions (to reach the same AXT final concentration, 1 μM) for 30 min, 1 h, 3 h, or 24 h. Cells were then fixed using 2.5% glutaraldehyde for 4 min and kept in phosphate buffer saline (PBS, Gibco Life Technologies) and stored at 4 °C until Raman imaging, or fixed using 4% paraformaldehyde for 10 min and proceeded to staining before fluorescence imaging.

MTS [3-(4,5-dimethylthiazol-2-yl)-5-(3-carboxymethoxyphenyl)-2-(4-sulfophenyl)-2H-tetrazolium] cell viability assay was carried out to assess the cytotoxicity of the used liposomes and free and encapsulated AXT. HAoEC were seeded on plastic-bottom 96-well plates with clear frames and were cultured in a complete EGM-2MV medium (Lonza, Basel, Switzerland) with the same supplementations as previously mentioned. All cells were incubated in a 37 °C, 5% CO2/95% air-humidified cell culture incubator. Cells were either pre-treated with 10 ng/mL TNF-α (Sigma Aldrich) or kept in a fresh medium for 24 h. Afterward, cells (except for the control group) were treated with cell culture media containing 1 μM AXT (Sigma Aldrich), dilution of liposome solutions (to reach the same AXT final concentration, 1 μM), or empty (not containing AXT) liposomes for 24 h. After the treatment, cells were washed, and according to the manufacturer’s instructions, 20 μL of MTS (CellTiter 96 AQueous One Solution Cell Proliferation Assay, Promega, USA) labeling reagent was added to each well, and plates were incubated at 37 °C for 2 h. Following MTS incubation, the absorbance of the samples was measured using Spark 10M microplate reader (Tecan, Switzerland) at 490 nm.

No significant effect on the cells’ viability was observed in the groups of cells treated with AXT, neutral or cationic liposomes or AXT encapsulated in either when used in the same concentrations or maximum incubation time (24 h) used in the manuscript (Online Resource, Fig. S1). Therefore, it was concluded that the used treatments were not cytotoxic at the conditions (concentrations and incubation times) used in the study.

### Raman imaging

Raman imaging was carried out using a WITec alpha 300 confocal Raman imaging system (WITec GmbH, Ulm, Germany) equipped with an ultra-high-throughput screening (UHTS) 300 spectrograph, a charge-coupled device (CCD) detector (Andor, DU401A-BV-352), and a × 60 water immersion objective (Nikon Fluor, NA = 1.0). An air-cooled solid-state laser with an excitation wavelength of 532 nm was used to excite the samples. Each measurement was done twice; once with a laser power at the cell samples of 3 mW (low laser power) and once with a laser power of 30 mW (high laser power). Low laser power measurements were done using a 0.5 s integration time and high laser power measurements were done using a 0.2 s integration time; a 0.5 μm step size was used for both.

### Staining and fluorescence imaging

Fixed HAoEC were permeabilized using 0.1% Triton-X 100 solution in PBS for 5 min, washed and blocked using 5% normal goat serum. Afterward, samples were incubated with ICAM-1 Monoclonal Antibody (Cat. No: MA5407, Invitrogen) (1.0 mg/mL, diluted 1:250) at 4 °C overnight. Then, they were washed with PBS and incubated with Alexa Fluor 647 goat-anti-mouse (Jackson Immunoresearch Laboratories, 1:300) in the dark at room temperature for 30 min, and then washed with PBS. BODIPY 493/503 (Invitrogen, 4 μg/mL) was used according to the manufacturer’s instructions to stain neutral lipids. Finally, Hoechst 33342 (Cat. No: H3570, Thermo Scientific) (10 mg/mL, diluted 1:1000) was used to stain cell nuclei for 10 min in the dark. After washing, cells were kept in PBS and imaged immediately. The confocal quantitative image cytometer (CQ1, Yokogawa) was used for fluorescence imaging.

### Data analysis

Raman spectra obtained from all samples were processed by a routine cosmic ray removal and were baseline corrected using auto-polynomial of degree 3 (WITec Project Plus software). Cluster analysis (CA) was performed with the k-means method (k-means cluster analysis, KMCA) using the Manhattan distance (WITec Project Plus software) utilized to obtain averaged spectra of the lipids classes. Origin Pro 2022 was used for normalizing and presenting the spectra.

ImageJ (National Institutes of Health; http://rsbweb.nih.gov/ij/) and Columbus Image Data Storage and Analysis System (Perkin Elmer) were used for the processing of the fluorescence images. The total number of cell nuclei (based on Hoechst staining), the number of lipid droplets (LDs), and the average fluorescence intensity of ICAM-1 (calculated using Columbus software from the total area of the cells) were calculated. To determine the statistical significance, a two-sample t-test was used (Origin Pro, 2022).

## Results and discussion

### AXT-loaded liposomes characterized by Raman spectroscopy

**Fig. 1:**
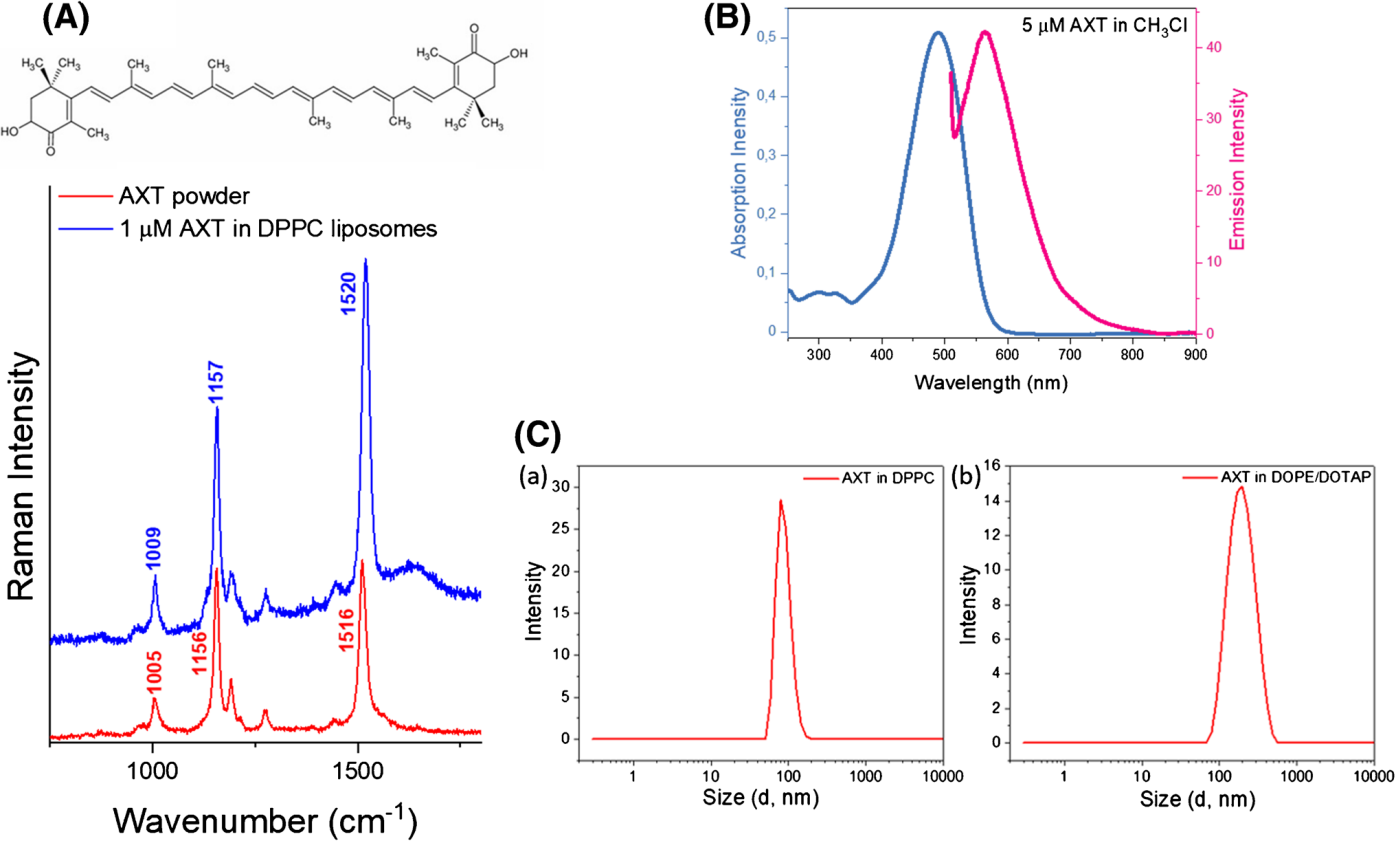
Characterization of astaxanthin-loaded liposomes**. A** Structure and Raman spectra of AXT powder (red) and in DPPC liposomes (blue). **B** Absorption and emission spectra of AXT in CH_3_Cl. **C** Dynamic light scattering (DLS) results of (a) DPPC liposomes (85 ± 10 nm) and (b) DOTAP-DOPE liposome (185 ± 15 nm), number of experiments (*n* = 3)

Two different types of large unilamellar vesicles (LUVs) of diameter 100 nm were used: neutral liposomes of DPPC lipid (1.3623 mM in PBS), and cationic liposomes comprised of DOTAP (0.2719 mM) and DOPE (0.27726 mM in PBS). Both types of LUVs were loaded with AXT at the same concentration (10 μM). It has been previously shown that there is a slight shift in the C=C stretching Raman band of AXT from ca. 1516 cm^−1^ for the solid state to ca. 1520 cm^−1^ for AXT dissolved in lipids [26]. The Raman spectra in Fig. 1A demonstrate a similar shift in the mentioned band between AXT powder and when it is encapsulated in DPPC liposomes. As shown in Fig. 1B, AXT absorbs light in the visible range coinciding with the 532 nm laser, which is usually used for cell measurements, and thus offers pre-resonance enhancement noticeable in the Raman spectrum of AXT. Specifically, resonantly enhanced Raman bands of AXT are observed at ca. 1009, 1159, and 1520 cm^−1^ corresponding to CH_3_ group wagging, C–C stretching, and C=C stretching vibrations, respectively.

### Time-dependent uptake of AXT and AXT-loaded liposomes by EC

**Fig. 2:**
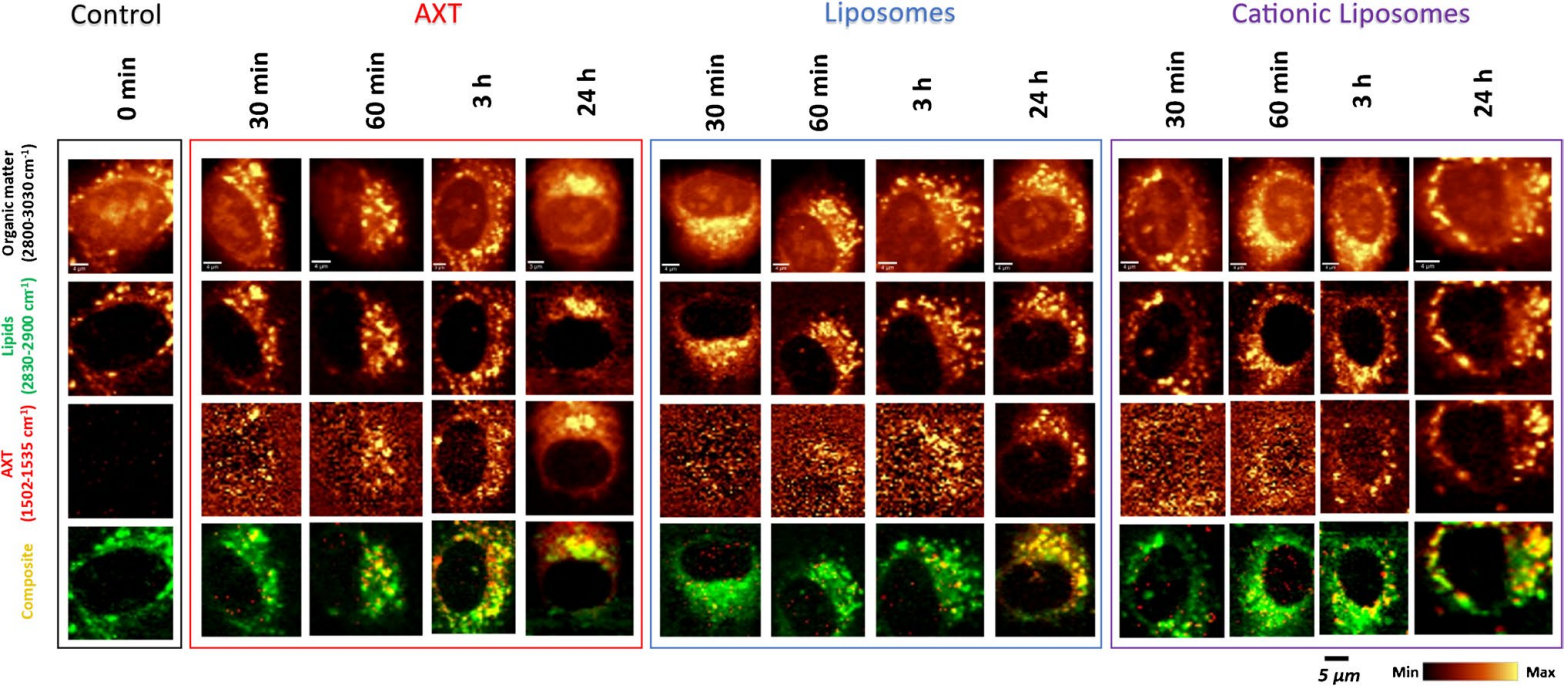
EC uptake of AXT-loaded liposomes studied by Raman imaging. Raman images of HAoEC, control, and cells incubated with AXT, AXT-loaded neutral or cationic liposomes, (for 30 min, 1, 3, and 24 h) obtained by the integration of the Raman bands over the selected spectral regions: 3030–2800 cm^−1^ (C–H stretching), 2900–2830 cm^−1^ (lipids), 1535–1502 cm^−1^ (astaxanthin), and composite images showing the distribution of lipids (green), AXT (red), and the overlay in yellow. Raman imaging of all samples was performed once with low laser power (3 mW) to detect AXT Raman bands and once with high laser power (30 mW) to detect bands associated with lipids

After EC incubation with AXT and AXT-loaded neutral or cationic liposomes using different incubation times, Raman spectra from AXT within the cells were visible only after 30 min of incubation. As expected, AXT was present in the cytoplasm of cells, specifically in the lipid-rich cellular organelles, i.e., LDs and endoplasmic reticulum (ER). As shown in Fig. 2, AXT accumulation in cellular lipids was time dependent. At shorter incubation times (30 and 60 min), AXT was found in lipid-rich areas in the cytoplasm but did not colocalize with all LDs of the cell. Starting from 3 h of incubation, AXT distribution demonstrates a clear colocalization with lipids in EC, determined by comparing the Raman maps of AXT (integration of the Raman spectra around 1520 cm^−1^) and lipids (integration of the Raman spectra around 2865 cm^−1^).

Subtle changes in the uptake and distribution of free AXT compared to that of encapsulated AXT were identifiable. For instance, it was noticeable that positively charged liposomes showed an enhanced uptake by HAoEC, demonstrated by a higher intensity of AXT marker bands, compared to neutral liposomes and free AXT. A similar effect has been previously reported in human corneal epithelial cells (HCECs) [9].

### Effect of liposomal encapsulation on AXT anti-inflammatory activity in EC

**Fig. 3:**
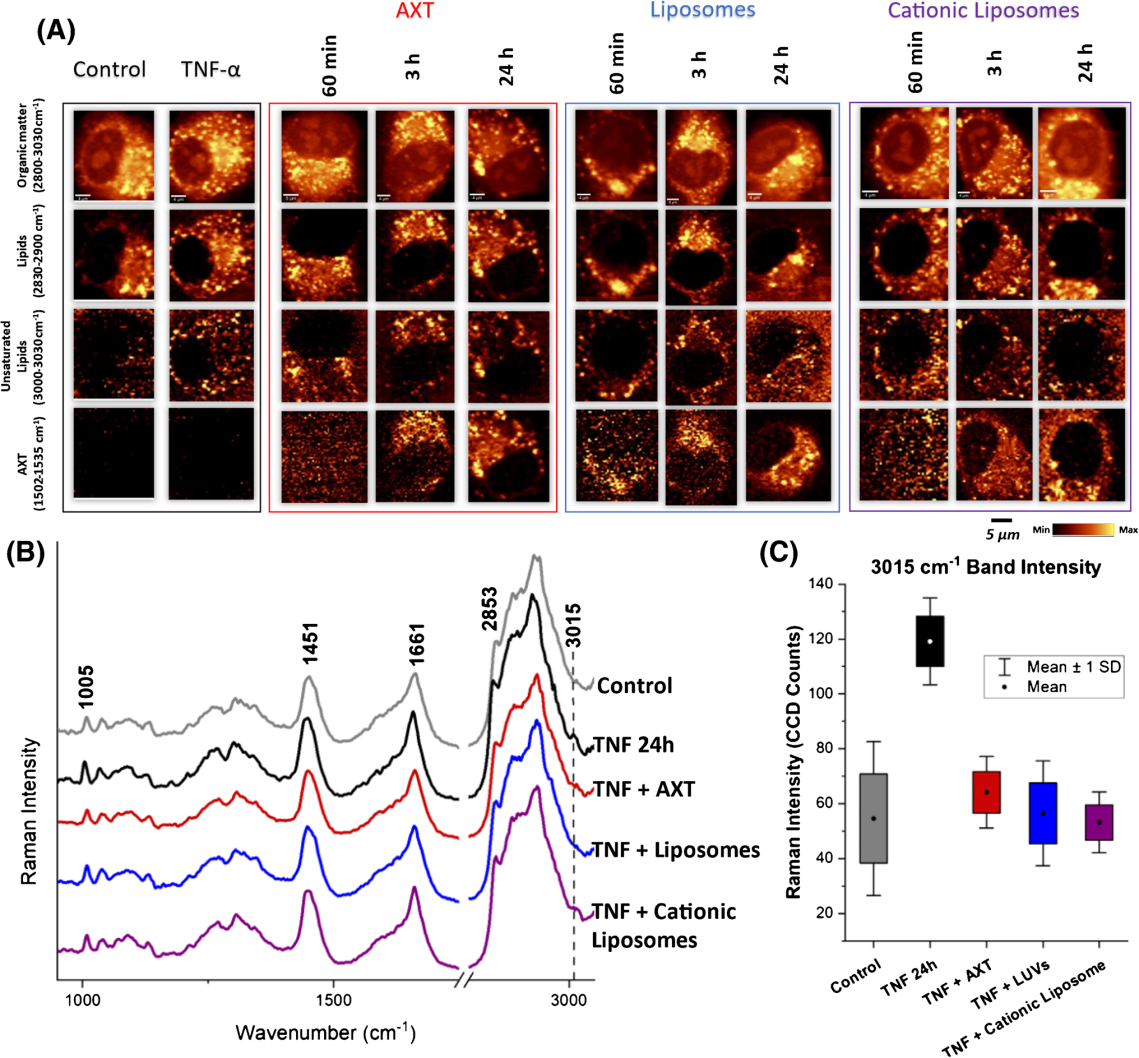
The effects of free and encapsulated AXT on activated EC lipids studied by Raman imaging. **A** Raman images of HAoEC, other than the control group; all the presented groups were pre-incubated with TNF-α for 24 h and then incubated with AXT, AXT-loaded  neutral or cationic liposomes, (for 1, 3, or 24 h). Pseudocolor images obtained by the integration of the Raman bands over the selected spectral regions: 2800–3030 cm^−1^ (C–H stretching), 2830–2900 cm^−1^ (lipids), 3000–3030 cm^−1^ (unsaturated lipids), and 1502–1535 cm^−1^ (AXT). Raman imaging of all samples was performed once with low laser power (3 mW) to detect AXT Raman bands and once with high laser power (30 mW) to detect bands associated with lipids. **B** Averaged Raman spectra of the lipid class of the control (grey), TNF-α pre-treated group (black), TNF-α pre-treated then incubated with AXT (red), AXT-loaded neutral liposomes (blue), and cationic liposomes (purple). **C** Quantification of Raman intensity of the band at 3015 cm^−1^ in different groups. *n* = 6 cells in each group, 3 independent experiments were performed

Both the number and the composition of LDs in EC have been previously shown to change when EC are activated using TNF-α [22]. This same effect can be seen in Fig. 3A; when control HAoEC is compared with TNF-α pre-treated group, a higher number of LDs are observed. Moreover, LDs in the TNF-α pre-treated group consist mostly of unsaturated lipids, evidenced by a higher intensity of the Raman band at ca. 3015 cm^−1^ (associated with the stretching modes of =C–H), which is characteristic of unsaturated lipids.

Following EC activation, cells were incubated with AXT, AXT-loaded neutral liposomes, or AXT-loaded cationic liposomes for 1, 3, or 24 h. Compared to the control, cells that were pre-incubated with TNF-α (10 ng/mL) for 24 h contained more LDs rich in unsaturated lipids, as shown in the third row (Fig. 3A). The prevalence of unsaturated LDs in inflamed cells that were later treated with AXT, either free or encapsulated, was shown to decrease, highlighting the effect of AXT in both free and encapsulated forms to attenuate the impact of TNF-α pre-treatment on EC lipid composition. Furthermore, encapsulated AXT in neutral or cationic liposomes showed a stronger effect on EC lipid unsaturation compared to free AXT. K-means cluster analysis (KMCA) was utilized to extract the spectra of the class of lipids in each cell. By comparing the averaged lipid spectra of each group, shown in Fig. 3B, a twofold increase in the intensity of the unsaturated lipids band at ca. 3015 cm^−1^ in the group of cells subjected to TNF-α pre-treatment was observed. This band was shown to decrease following incubation with AXT and AXT-loaded liposomes, showcasing the capability of Raman imaging to detect AXT accumulation and the uptake of free and encapsulated AXT. Moreover, the data indicated changes in the lipid composition of the EC in the cases of inflammation and anti-inflammatory treatment with free or encapsulated AXT.

**Fig. 4:**
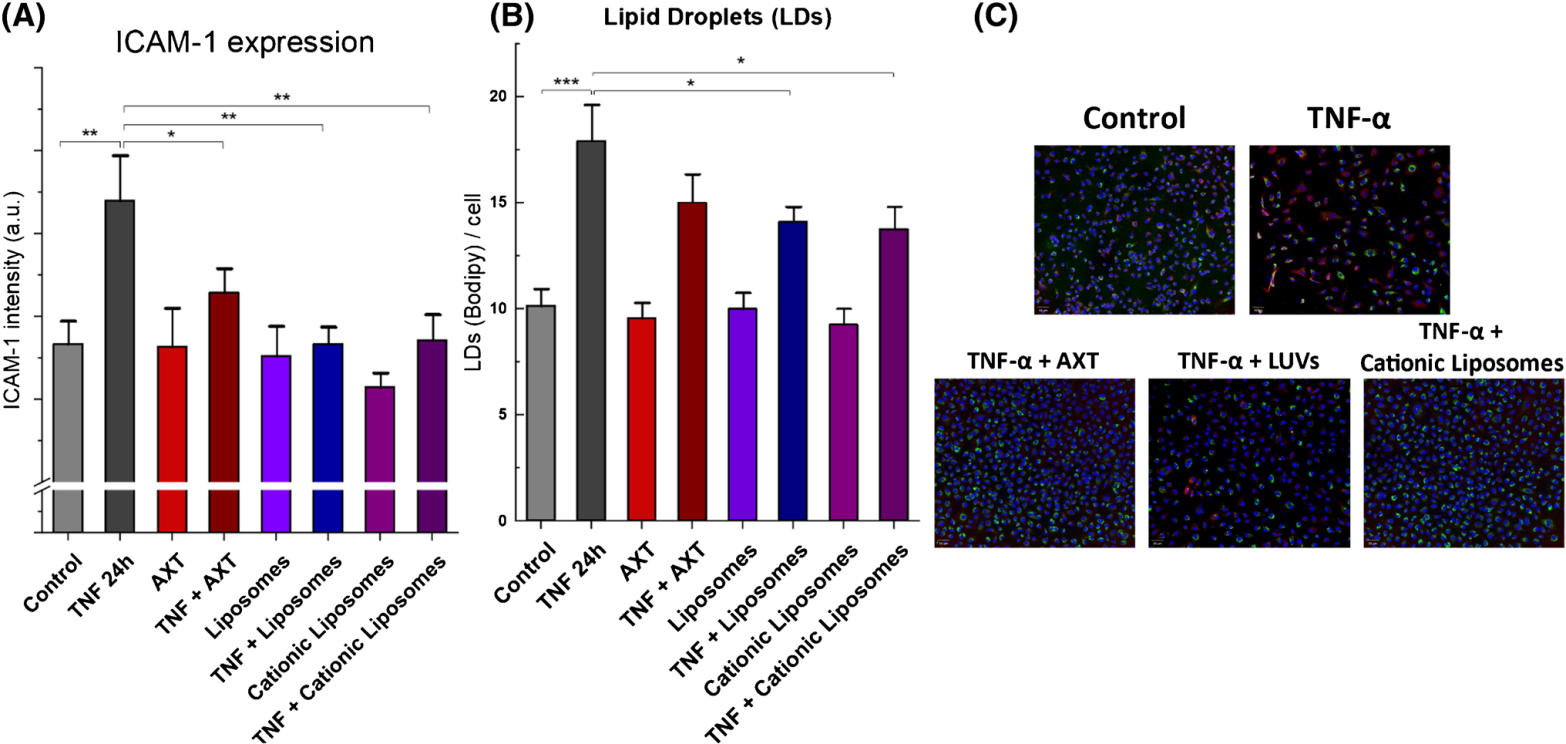
Fluorescence-based quantification of free and encapsulated AXT anti-inflammatory effects. **A** ICAM-1 expression and **B** lipid droplets (LDs) per cell of control and TNF-α pre-treated groups that were later treated with AXT, AXT-loaded neutral liposomes, or AXT-loaded cationic liposomes. The results are presented as the means + SD from 3 independent experiments, * *P* < 0.05, ** *P* < 0.01, and *** *P* < 0.001. **C** Representative fluorescence images of HAoEC, showing nuclei (Hoechst stained, blue), lipids (BODIPY stained, green), and ICAM-1 (red). Scale bars equal 50 μm

To quantify the anti-inflammatory effect and changes in LD content induced in activated EC by AXT, AXT-loaded neutral and cationic liposomes, fluorescence imaging was utilized as a reference method. Expression of ICAM-1, a well-known marker of EC inflammation, was compared among the different groups. A significant ICAM-1 overexpression was seen following activation with TNF-α compared to control, reflected in the relative ICAM-1 expression of the TNF-α pre-treated cells which was 119.2 ± 4.6% of the control (Fig. 4A). This overexpression was significantly decreased to the control level when cells were treated with free or encapsulated AXT. Moreover, this anti-inflammatory effect is stronger in cells treated with AXT-loaded liposomes, as the relative ICAM-1 expressions of activated EC treated with free AXT, AXT in neutral liposomes, and AXT in cationic liposomes were 106.6 ± 2.4%, 99.9 ± 1.8%, and 100.5 ± 2.6% of the control, respectively.

A significant increase in the number of LDs per cell in the group of cells incubated with TNF-α was observed (Fig. 4B). While the number of LDs was decreased after treatment with free AXT, this effect was not significant. On the other hand, when activated EC were treated with encapsulated AXT in neutral or cationic liposomes, the number of LDs per cell decreased by 22% and 24%, respectively, compared to TNF-α incubated cells that were not subjected to anti-inflammatory treatment.

The results of two complementary methods, Raman and fluorescence microscopies, clearly indicate that the anti-inflammatory effects of AXT on EC can be enhanced by encapsulating AXT both in neutral and positively charged liposomes, specifically in terms of decreasing lipid unsaturation and lowering ICAM-1 overexpression. Moreover, while free AXT does not significantly decrease the number of LDs in inflamed EC, encapsulated AXT lowered the numbers of LDs in inflamed EC significantly.

## Conclusions

This study examined the uptake of AXT encapsulated in neutral and positively charged liposomes by Raman microscopy with the support of fluorescence microscopy. Raman imaging provided insight into the uptake of free and encapsulated AXT by EC in healthy and inflamed states. EC incubated with TNF-α showed alterations in LD composition toward more unsaturated lipids, a feature previously linked to EC inflammation. Moreover, the anti-inflammatory effect of AXT-loaded liposomes has been demonstrated by Raman imaging, highlighting a decrease in the unsaturated lipids content in activated EC subjected to anti-inflammatory treatment.

Similarly, fluorescence imaging showed a significant anti-inflammatory effect of AXT-loaded neutral and cationic liposomes on activated EC, manifested by a substantial decrease in ICAM-1 overexpression and the number of LDs per cell. While free AXT treatment resulted in a significant decrease of ICAM-1 expression, it did not have as significant an effect on LDs as encapsulated AXT.

It is worth noting that it is challenging to accurately quantify the intracellular AXT content using the presented Raman microscopy–based method due to the resonance effect of AXT. Nevertheless, semi-quantitative information on the AXT distribution and comparative assessment in different conditions can be acquired. The presented method is also rather time consuming due to the longer image acquisition time and the need to image each sample twice (once with lower and once with higher laser powers) because of AXT sensitivity to higher laser power. On the other hand, the use of Raman imaging to analyze AXT uptake and effects on EC lipids could be done in a label-free manner, eliminating the undesirable effects of dyes used for fluorescence-based detection (such as the risk of photobleaching, the biological effects of the dyes affecting the results, the need for the additional steps of blocking, staining, and washing). Furthermore, the utilization of techniques such as stimulated Raman scattering (SRS) microscopy would allow much faster detection of cellular uptake and effects of various molecules in future studies.

In conclusion, encapsulating AXT in neutral or positively charged liposomes enhanced AXT’s anti-inflammatory effects, in terms of decreasing EC lipid unsaturation and ICAM-1 overexpression, and reduced the number of LDs in inflamed EC, while free AXT did not significantly have an effect decreasing the number of LDs. Raman imaging provided information on AXT uptake and accumulation and changes in EC lipid composition, whereas fluorescence imaging allowed quantification of the anti-inflammatory effects of encapsulated AXT.

## Supplementary information

ESM 1 (DOCX 27 kb)

## Data Availability

The datasets generated during and/or analyzed during the current study are available from the corresponding author on reasonable request.
