# Supplementary material for: Uptake and anti-inflammatory effects of liposomal astaxanthin on endothelial cells tracked by Raman and fluorescence imaging
Source: Mikrochim Acta. 2023 Jul 27;190(8):332. doi: 10.1007/s00604-023-05888-8 (PMC10374751; doi:10.1007/s00604-023-05888-8)
Supplement: Supplementary file 1 — (DOCX 27 kb) [file 604_2023_5888_MOESM1_ESM.docx]

**Electronic Supporting Information**

**Uptake and anti-inflammatory effects of liposomal astaxanthin on endothelial cells tracked by Raman and fluorescence imaging**

Microchimica Acta

Basseem Radwan^1,2^, Amrutha Prabhakaran^3^, Stefano Rocchetti^1^, Ewelina Matuszyk^1^, Tia E. Keyes^3^ and Malgorzata Baranska^1,2*^

^1^ Jagiellonian Centre for Experimental Therapeutics (JCET), Jagiellonian University, 14 Bobrzynskiego Str., 30-348 Krakow, Poland.

^2^ Faculty of Chemistry, Jagiellonian University, 2 Gronostajowa Str., 30-387 Krakow, Poland.

^3^ School of Chemical Sciences and National Centre for Sensor Research, Dublin City University, Dublin 9, Ireland.

*Correspondence: [m.baranska@uj.edu.pl](mailto:m.baranska@uj.edu.pl)

**Fig. S1 Cytotoxicity Assessment of AXT, liposomes and lipoplexes on HAoEC.** Cell viability (percentage of viable cells based on MTS absorbance values) of control, and TNF-α (10 ng/ml, 24h) pre-treated groups that were later treated with AXT (1 µM), empty liposomes, empty lipoplexes, AXT-loaded liposomes or AXT-loaded lipoplexes (to reach the same AXT final concentration of 1 µM, 24h). The results are normalized to the Control and presented as the means + SD.
